# Finite element analysis of mechanical stress in a cementless tapered-wedge short stem in the varus position

**DOI:** 10.1186/s13018-024-04856-z

**Published:** 2024-07-01

**Authors:** Takahiro Maeda, Osamu Obayashi, Muneaki Ishijima, Taichi Sato, Yoshiro Musha, Hiroyasu Ikegami

**Affiliations:** 1 Department of Orthopedic Surgery, Toho University Graduate School of Medicine, 5-21-16 Omorinishi, Ota-ku, Tokyo, 143-8540 Japan; 2 Department of Orthopedic Surgery (Ohashi), School of Medicine, Toho University, 2-22-36 Ohashi, Meguro-ku, Tokyo, 153-8515 Japan; 3 Department of Orthopedic Surgery, Juntendo Shizuoka Hospital, Nagaoka 1129, Izunokuni 410-2295, Shizuoka, Japan; 4 Department of Medicine for Orthopedic and Motor Organ, Juntendo University Graduate School of Medicine, 2-1-1, Hongo, Bunkyo-ku, Tokyo, 113-8421 Japan; 5 Department of Advanced Machinery Engineering, School of Engineering, Tokyo Denki University, 5 Senju Asahi-cho, Adachi-ku, Tokyo, 120-8551 Japan

**Keywords:** Mechanical stress, Varus position, Malalignment, Finite element analysis, Stress shielding, Cortical hypertrophy, Total hip arthroplasty, Tapered-wedge stem, Short stems, Gruen’s zone

## Abstract

**Background:**

In recent years, the use of tapered-wedge short stems has increased due to their ability to preserve bones and tendons. Surgical techniques occasionally result in a varus position of the stem, which is particularly pronounced in short stems. Although the varus position is not clinically problematic, there are reports of an increased incidence of stress shielding and cortical hypertrophy. Thus, we evaluated and examined the acceptable range of varus angles using finite element analysis.

**Methods:**

Patients diagnosed with osteoarthritis of the hip joint who had undergone arthroplasty were selected and classified into three types [champagne-flute (type A), intermediate (type B), and stovepipe (type C)]. Finite element analysis was performed using Mechanical Finder. The model was created using a Taperloc microplasty stem with the varus angle increased by 1° from 0° to 5° from the bone axis and classified into seven zones based on Gruen’s zone classification under loading conditions in a one-leg standing position. The volume of interest was set, the mean equivalent stress for each zone was calculated.

**Results:**

A significant decrease in stress was observed in zone 2, and increased stress was observed in zones 3 and 4, suggesting the emergence of a distal periosteal reaction, similar to the results of previous studies. In zone 2, there was a significant decrease in stress in all groups at a varus angle ≥ 3°. In zone 3, stress increased from ≥ 3° in type B and ≥ 4° in type C. In zone 4, there was a significant increase in stress at varus angles of ≥ 2° in types A and B and at ≥ 3° in type C.

**Conclusion:**

In zone 2, the varus angle at which stress shielding above Engh classification grade 3 may appear is expected to be ≥ 3°. Distal cortical hypertrophy may appear in zones 3 and 4; the narrower the medullary cavity shape, the smaller the allowable angle of internal recession, and the wider the medullary cavity shape, the wider the allowable range. Long-term follow-up is required in patients with varus angles > 3°.

## Background

Various types of implants are used in total hip arthroplasty (THA). The use of short-stem implants has increased in recent years due to greater bone preservation, better proximal load transfer, less invasive surgery, and ease of use of the anterior approach [1–3]. The postoperative stress distribution in the femur changes significantly after stem placement as the load is transmitted to the femur through the stem. Bone density in the proximal femur decreases in the early postoperative period after THA, and remodeling occurs to accommodate the new load. Stresses in the proximal femur are low, and bone atrophy occurs; in the long term, bone atrophy may lead to peri-implant fractures and stem instability [4–6].

Short stems transmit stress more easily proximally than standard stems, and many surgeons have reported good results with short stems [7, 8]. One disadvantage of short stems is that they tend to be inserted with misalignment. The stem should be inserted horizontally along the bony axis. However, depending on the skill of the surgeon, implant design, and approach technique, the stem may be in the varus or valgus position [9]. Short stems, in particular, are prone to the varus position, and peri-stem bone reactions due to the varus position have been reported [10].

We used finite element analysis to investigate how stress transfer changes with the degree of varus position, especially for short stems that are prone to the varus position. This study aimed to evaluate the stresses on the femur as the varus angle increases and to investigate the varus angle at which peri-stem bony reactions can occur.

Therefore, in this study, we used preoperative computed tomography (CT) images of patients undergoing surgery to examine the change in stress when the stem was tilted in increments of 1° varus angle.

## Methods

This study included cases of hip osteoarthritis that underwent joint replacement surgery at Juntendo University Shizuoka Hospital between April 1, 2018, and March 31, 2021. The patients who underwent preoperative CT were classified into three femoral types based on Noble’s Canal Flare Index (CFI), with CFI < 4.7 being the champagne-flute type (type A), CFI < 3 being the stovepipe type (type C), and CFI between the two being intermediate type (type B). As 10 cases with the champagne-flute type were indicated, 10 cases of types B and C were also randomly selected [11].

Since only two male patients were included in type A and the other patients were female, we excluded the male patients to ensure homogeneity, as being male has been identified as a risk factor for proximal stress shielding in previous studies, and other reports have consistently focused on female patients (Table 1) [9, 12, 13]. 

**Table 1:** Patient demographics

	Age (years)	Sex	Side (right/left)	CFI
Type A	63 ± 9.9	Female	4 / 4	5.19 ± 0.35
Type B	71 ± 11.9	Female	8 / 2	3.56 ± 0.26
Type C	73 ± 14.6	Female	5 / 5	2.49 ± 0.14

*Values are mean

Type A (champagne-flute), Type B (intermediate), Type C (stovepipe).

CFI, canal flare index; Side femoral affected side

**Fig. 1:**
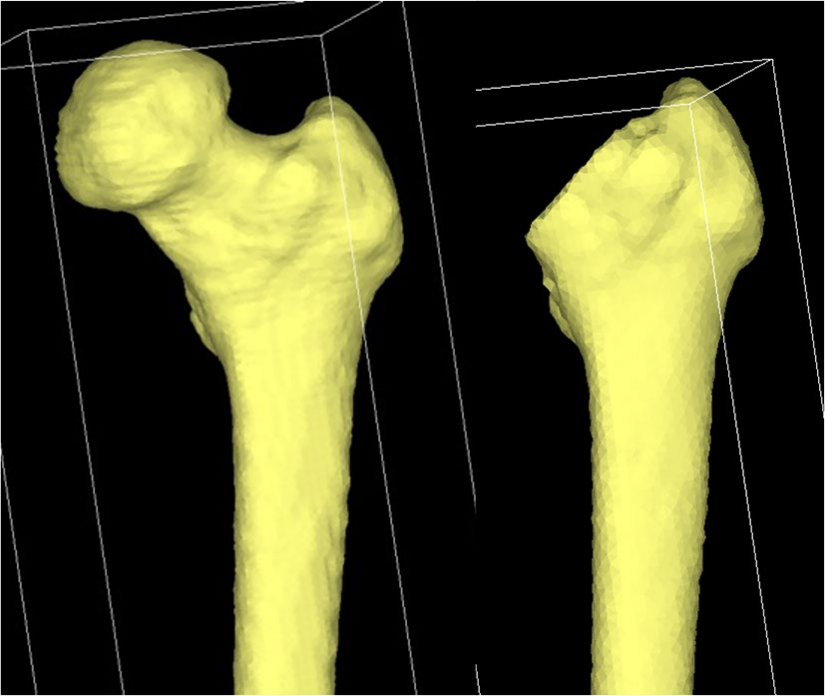
3D bone model. The DICOM data of the preoperative femur CT were read to extract the contour of the femur and create a 3D bone model using tetrahedral elements. The femur was osteotomized approximately 10 mm from the lesser trochanter

Finite element analysis was performed using Mechanical Finder ver. 10.0 (Research Center for Computational Mechanics, Japan). CT DICOM data (Discovery CT750 HD; GE Medical Systems, Milwaukee, WI, USA) were used to create a finite element model. One millimeter slice thickness of the affected femur was obtained from the preoperative CT of the extracted case. The femur contour was extracted, and a 3-D finite element model was created using tetrahedral elements. The stems were subjected to Taperloc microplasty (Zimmer Biomet Holdings Inc., Indiana, USA). The stem used was a cementless, tapered-wedge stem, which is shorter than the standard stem. Stem contours were captured using a 3D scanner (ATOS Core Education, GOM, Germany). The contours and coordinate axes were corrected, converted to stereolithography data, and imported into Mechanical Finder. The femoral model was osteotomized approximately 10 mm proximal to the lesser trochanter. The stem was placed through the proximal metaphyseal bone axis, and stem anteversion was performed in the direction of the femoral neck bone axis [14] (Fig. 1).

The stem was placed by the same examiner, and the appropriate size was determined to be a proximal metaphyseal fit, with the medial and lateral sides of the stem fitted into the bone cortex [15].

A total of 168 bone models were created: 48 (with 8 examples) for type A, 60 (with 10 examples) for type B, and 60 (with 10 examples) for type C; the varus angle increased by 1° from 0° to 5° from the bone axis.

**Fig. 2:**
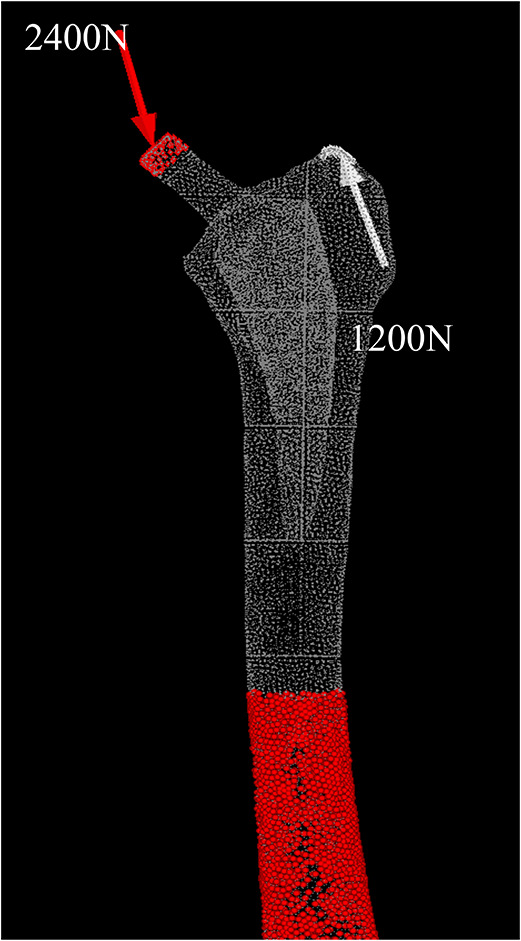
Analysis conditions. The load restraint conditions were set assuming a one-leg standing posture, with 2,400 N at the stem head from 15° proximal medial to the femoral axis and 1,200 N at the abductor muscles from 20° distal lateral to the femoral axis

Load restraint conditions were set assuming a one-leg standing position, with a joint reactive force of 2,400 N exerted by the weight of the body on the femoral head or prosthetic head at an angle of 15° proximal medial relative to the femoral axis, and a 1,200 N force generated by the abductor muscles exerted at an angle of 20° distal lateral relative to the greater trochanter [16, 17] (Fig. 2).

**Fig. 3:**
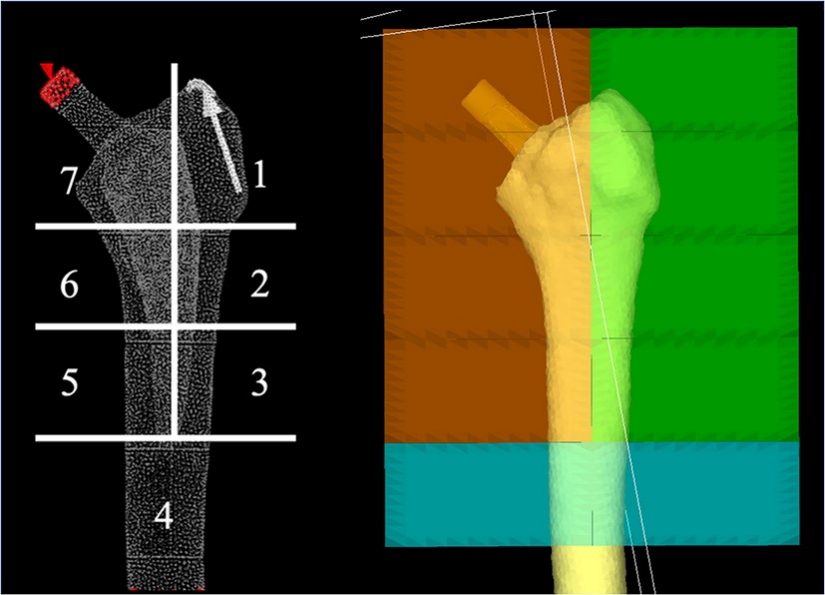
Segmentation of the finite element models of the femur according to Gruen’s zones

Young’s modulus based on CT values (Hounsfield units) was set for each element using the conversion formula of Keyak et al. [18] for femoral material properties to reflect the differences in bone density in each case. Young’s modulus of the stem was set at 108.85 GPa. The Poisson’s ratio of the femoral material was set to 0.40, and the stem was made of titanium (Ti-6Al-4 V) with a Poisson’s ratio of 0.28 [18]. The stem and femur were assumed to share a nodal point and completely adhere to each other. Seven zones were classified based on Gruen’s zone classification, the volume of interest was set, and the mean value of the equivalent stress for each zone was calculated [19] (Fig. 3).

EZR software (version 4.2.2) was used for the statistical analysis [20]. Multiway analysis of variance (ANOVA) was applied for between-group comparisons, and repeated-measures analysis of variance, and multiple comparisons (Bonferroni method) were applied for within-county comparisons at a significance level of 5%.

## Results

**Fig. 4:**
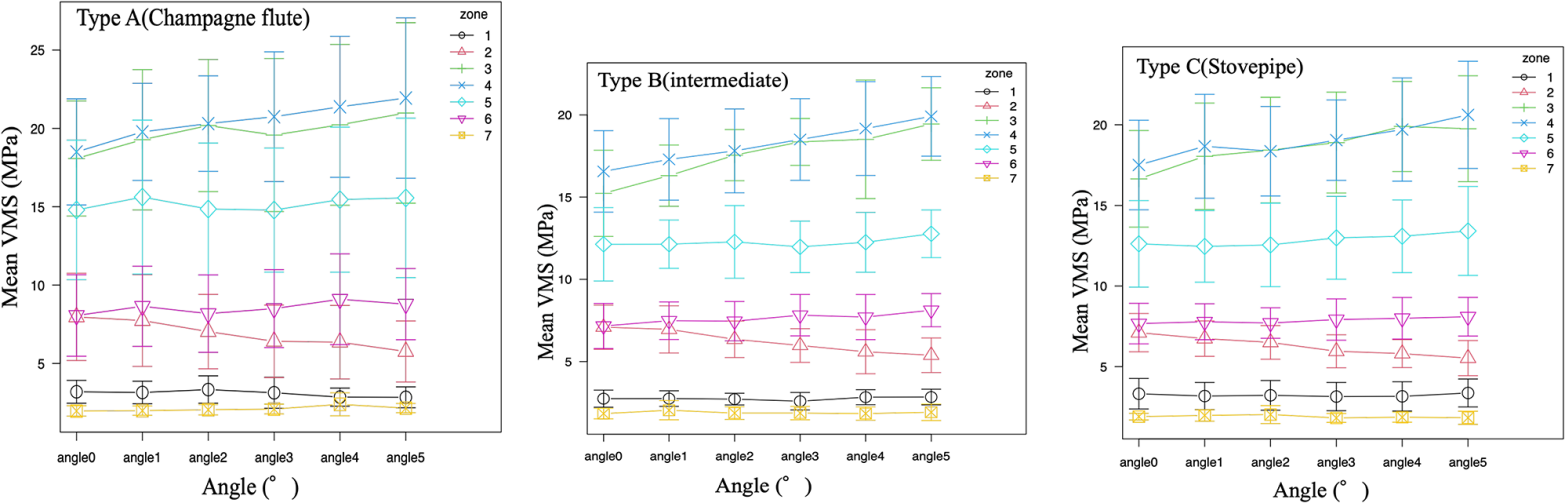
Change in von Mises stress values in Gruen’s zone with increasing varus angle

First, a comparative study of the average stress in each zone with increasing internal warping angle was conducted. In zone 2, all femur types showed a significant decrease in stress from a varus angle of 3°, whereas in zone 3, femur types B and C showed significant increases in stress from varus angles of 3° and 4°, respectively. In zone 4, there was a significant increase in stress from a varus angle of 2° in femurs A and B and from a varus angle of 3° in type C (Table 2; Fig. 4).

**Table 2:** Varus angles at which a significant increase or decrease in von Mises stress began to occur in the Gruen zone

Zone	Von Mises stress	Type A	Type B	Type C
		Varus angle (°)		
Zone 2	Decrease	3°≤ (*P* = 0.001)	3°≤ (*P* = 0.046)	3°≤ (*P* = 0.007)
Zone 3	Increase	(*P* = 0.077)	3°≤ (*P* = 0.003)	4°≤ (*P* = 0.002)
Zone 4	Increase	2°≤ (*P* = 0.017)	2°≤ (*P* = 0.004)	3°≤ (*P* = 0.001)

**Fig. 5:**
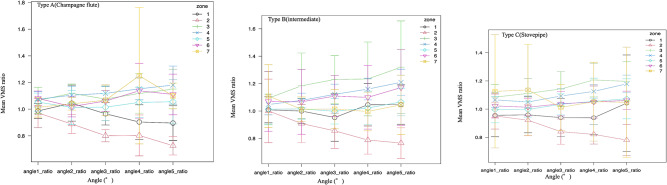
Change in the von Mises stress ratio in Gruen’s zone with increasing varus angle. von Mises stress ratio; von Mises stress values of varus angle 1°, 2°, 3°, 4°, 5° / von Mises stress values of varus angle 0°

Second, since the quality of each bone was different, we examined the rate of change instead of the stress value. In zone 2, there was a significant decrease in stress in femur types A and C from a varus angle of 3°, a significant increase in stress in femur type B from a varus angle of 3°, and a significant increase in stress in femur types A and C from a varus angle of 4°. In zone 4, there was a significant increase in stress in femur type B from an angle of 4 ° and in femur type C from a varus angle of 5° (Table 3; Fig. 5).

**Table 3:** Change in von Mises stress ratio concerning the 0° varus angle in Gruen’s zone with increasing varus angle

Zone	Von Mises stress	Type A	Type B	Type C
		Varus angle (°)		
Zone 2	Decrease	3°≤ (*P* = 0.026)	4°≤ (*P* = 0.022)	3°≤ (*P* = 0.002)
Zone 3	Increase	(*P* = 0.118)	3°≤ (*P* = 0.027)	4°≤ (*P* = 0.034)
Zone 4	Increase	(*P* = 0.483)	4°≤ (*P* < 0.001)	5°≤ (*P* = 0.012)

**Fig. 6:**
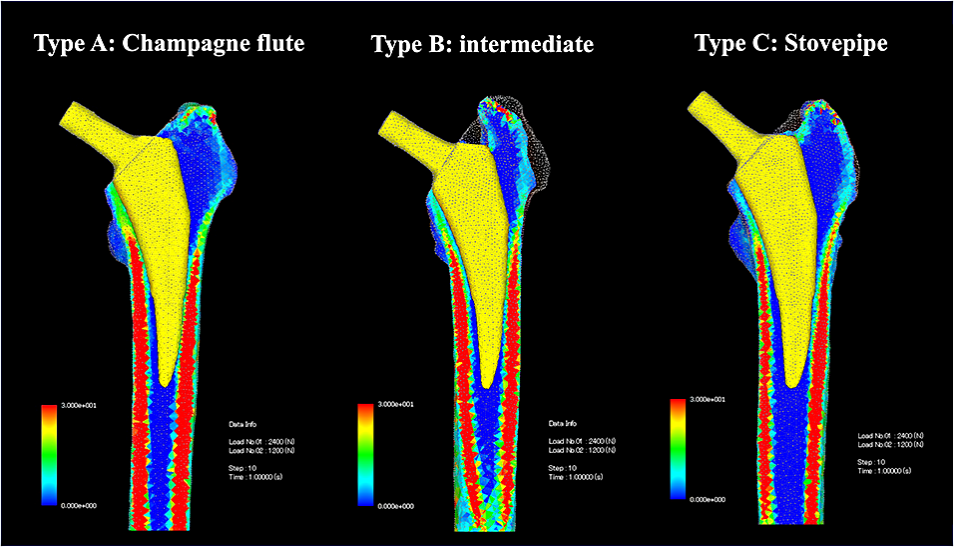
Stress distribution diagram when the stem is neutrally inserted. Good load transfer occurred distally from Gruen zones 2 and 6

**Fig. 7:**
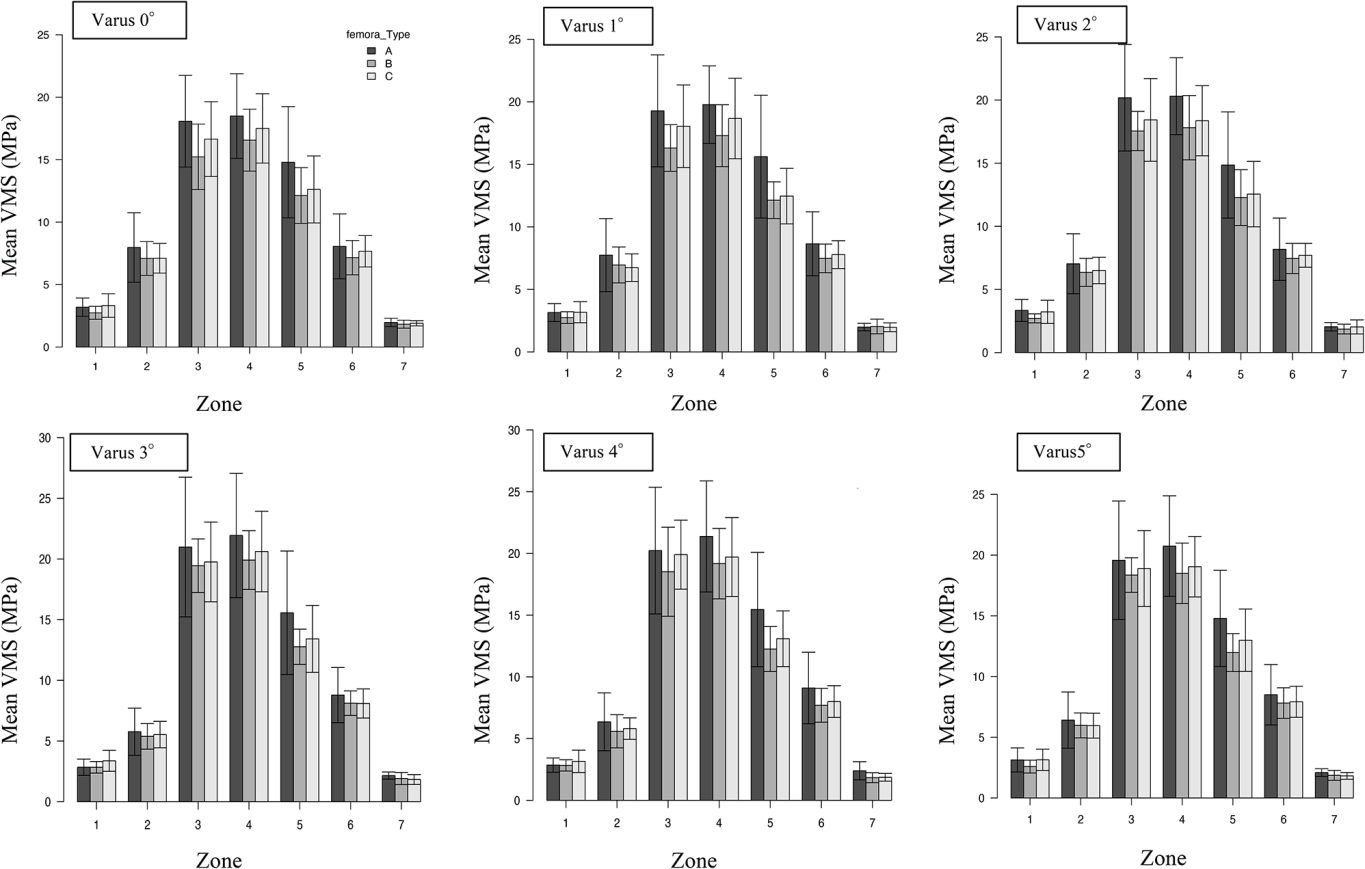
Comparison of stresses between femur types. The mean VMS values per zone for each angle are shown. Zone 4 shows the highest stress for all types, and type A (champagne flute) shows the highest stress value VMS: von Mises stress

Furthermore, we examined whether there was a significant difference in stress between the femurs in each zone at each angle. The stress was highest in zones 3 and 4 for all femur types, with type A having the highest stress, followed by types B and C (Table 3; Fig. 5). There was no significant difference in the stress in the zones owing to the change in the angle between the femur types (Figs. 6 and 7).

## Discussion

Recently, the use of tapered-wedge short stems has increased because of their ability to preserve bone and tendon. Barrington et al. [1] compared the postoperative results of the Taperloc standard stem and microplasty and found that both had good results, with low revision rates of 0.9%/1.0%. Molli et al. reported a lower intraoperative fracture risk of 3.1%/0.4% with Taperloc microplasty, indicating that the short stem is useful [2].

Depending on the surgical technique, implant design, and skill of the surgeon, the stem may be placed in the varus position. This is particularly true for short stems, which are not inserted into the distal medullary cavity but only into the proximal medullary cavity, thus increasing the degrees of freedom [10, 21, 22].

Although it has been reported that the varus position is not clinically problematic [10], Vresiovic et al. evaluated stem fixation and radiographic images at the time of surgery in patients who underwent THA revision for pain and found that 83% of the varus position cases had instability and a high revision rate, which was associated with clinical symptoms [23].

Short stems have been reported to have less stress loading around the stem than standard stems and the proximal loss of bone density caused by stem insertion is significantly less with short stems because force transmission is shifted more proximally [7, 8]. However, there are few reports of peri-stem bony reactions in short stems, although there are no comprehensive reports on such reactions. Evola et al. reported an increased risk of femoral cortical hypertrophy and pain associated with varus insertion [24]. Crawford et al. found distal femoral cortical hypertrophy in 74% of cases in Gruen zone 3 and 56% in zone 5, and Innmann et al. found cortical hypertrophy in 56% of cases, mostly in zones 3 and 5 [25, 26].

In the present study, there was a significant decrease in stress in zone 2 and increased stress in zones 3 and 4, suggesting the appearance of a distal periosteal reaction, similar to the results of previous studies. In the comparison of mean stress values, zone 2 showed a significant decrease in all groups at a 3° varus angle, and the acceptable varus angle for the appearance of SS above Engh classification grade 3 was expected to be 3° [27]. Kwak et al. [28] performed a mechanical finite element analysis using 12 healthy femoral models, with the stem and femur sharing a contact point. They reported that the varus model showed an increase in stress at the lateral interface compared to the intermediate insertion model, which was similar to the present study. Their study differed from the present study in that it was a comparison of intermediate and internal insertions using a healthy femoral model, but their findings were consistent with the results in the osteoarthritis cases used in the present study and are a contributing factor to the rationale of the study [28].

In zone 3, there was an increase from a 3° varus angle in type B and from a 4° varus angle in type C. In zone 4, there was a significant increase in stress from a 2° varus angle in types A and B and a 3° varus angle in type C. This suggests the appearance of cortical hypertrophy distally, with a narrower medullary lumen shape indicating a narrower tolerance range and a wider medullary lumen shape indicating a wider tolerance range.

Considering the differences in the bone quality of each patient, we also analyzed the rate of change in stress with increasing varus angle from 0° as a reference point. In zone 2, we observed a significant decrease in stress in all groups at a varus angle of ≥ 3°. In zone 3, the stress increased significantly from 3° for type B to 4° for type C, whereas in zone 4, the stress increased significantly from 4° for type B to 5° for type C. As mentioned above, the tolerance range increased with the medial cavity geometry.

Oba et al. [12] compared the stress distribution in the Accolade TMZF stem (tapered-wedge cementless stem) with different luminal geometries (cases with stem alignment < 3°) and analyzed three luminal types (champagne-flute, intermediate, and stovepipe). The authors reported that the stovepipe type tended to have a larger stem size, a stem contacting the cortex distally, and a decrease in stress proximally and that bone mineral density decreased in zone 2, although there was no significant difference in stress in Gruen’s zone 6 [12]. Cooper et al. also studied the postoperative radiographic osteointegration of Accolade TMZF stems and reported that the risk of proximal stress shielding and proximal osteointegration failure was associated with a small CFI (stovepipe type) and a large stem size [13]. Ishii et al. reported that osteointegration failure was more common in patients with a large CFI (champagne-flute type), which they attributed to distal fixation due to the narrow femoral marrow cavity in Asians, and concluded that both stovepipe and champagne-flute types should be treated with caution [29].

**Fig. 8:**
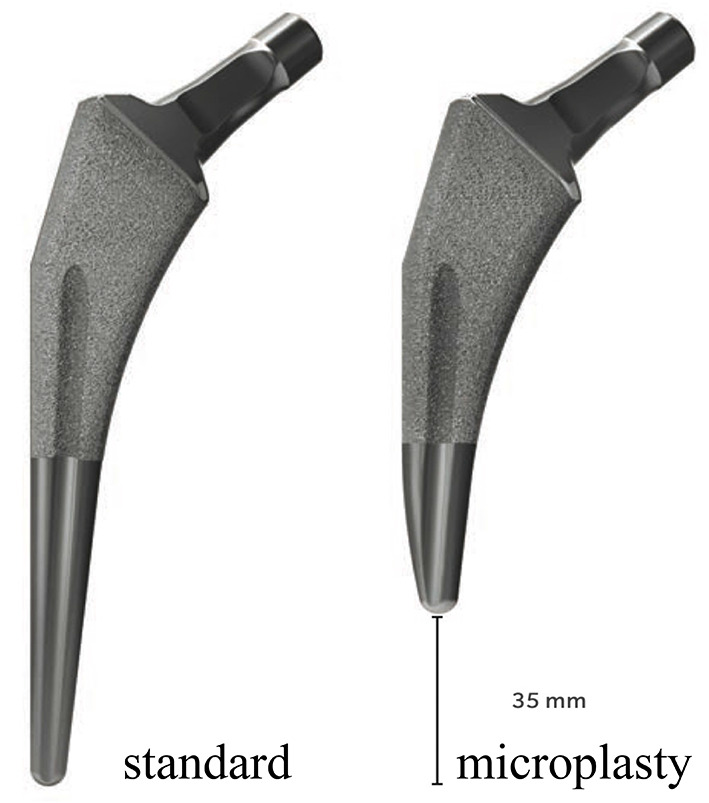
Taperloc stem (Zimmer Biomet Holdings, Inc., Indiana, USA). **(a)** Standard stem and **(b)** short stem (35 mm shorter than the standard stem)

In the present study, there was no change in the stress distribution in each medullary cavity when the insertion was parallel to the bone axis (0° varus angle), and there was no significant difference between the groups. However, the highest stresses were found in zone 4, and there was no change in the stress distribution from zones 2 and 6 distally. The reason for the lack of change in stress distribution due to the difference in the medullary cavity is that, unlike the stems used by Oba et al., Cooper et al., and Ishi et al., the stem shape is a reduced structure to avoid distal fixation, and the stem is not distally fixed, which is thought to be because it adapts to various medullary cavity configurations and transmits stress (Fig. 8).

Since an appropriate stem size was used in this study, it was believed that Taperloc microplasty could accommodate any luminal configuration and transmit stress firmly distally, at least if the proper size was selected in preoperative planning and the stem alignment was neutral. However, as in the studies by Oba et al. and Ishi et al., this study was limited to women only, and Cooper et al. found the male sex to be a risk factor. Therefore, the results of this study conducted on women may not be applicable to men, and caution is required when treating men. Further analysis of male patients is required. A narrow medullary space of this type showed a significant increase in distal stress with a 2° varus angle. Therefore, stem insertion should be performed more carefully in patients with a narrow medullary space.

This study has a few limitations. The main limitation of this study was that, in setting the boundary conditions, the femoral material was analyzed with a Poisson’s ratio of 0.40, the stem was made of titanium (Ti-6Al-4 V) with a Poisson’s ratio of 0.28, and the stem and femur were analyzed with a shared contact point and complete adherence between the stem and femur. The stems were proximally porous and distally smooth. To construct a realistic model, different friction coefficients must be set for each stem surface. This study might have been considered an osteointegrated model (stem and femur adhered), which may not be sufficient to be considered as a result of initial fixation. Although Oba et al. also mentioned this as a limitation of their study, they considered that assuming successful fixation of the cementless stem, finite element analysis may show characteristic stress distributions, even with a simplified finite element model [12]. Furthermore, they compared the results with bone mineral density (BMD) and stated that mechanical stress changes in bone remodeling are consistent with changes in BMD measured during the first postoperative year, which may cause bone loss even after the successful initial fixation of the stem. The results of our study are considered useful after initial fixation. Second, no comparison with actual cases or bone density evaluation was performed. Third, stem insertion in fragile bones, such as in femoral neck fractures, should be considered separately. Fourth, this study only examined the varus insertion of the stem in the coronal plane. In clinical practice, the stem is inserted valgus in the coronal plane, anteversion in the axial plane, or flexion/extension in the sagittal plane. In this study, we examined only varus insertion, which is common in clinical practice; however, to reproduce clinical practice, it is necessary to examine all combinations of anteversion and flexion/extension parameters. Obtaining an acceptable range of angles at which stress changes occur, which was the objective of this study, was difficult because of the large number of combinations.

Kaku et al. [7] examined the stresses in the stem after setting the angles as 2° internal/external, 3° flexion/extension, and 10° or 40° anteversion. They reported that the increase in stress around the stem is greater in extension than in flexion and that the lesser the anteversion, the greater the increase in stress. A limitation of this study was that only one femoral model was used, and the medullary cavity and bone quality were not considered [7]. It is also unclear if this result is significant as the paper does not include statistical analysis; however, changes in flexion/extension and anteversion may also cause changes in stress, which could change the results of this study. However, the results of our study, which considered the medullary cavity and were obtained in multiple patients, are considered reasonable. Fifth, as no comparison was made with healthy femurs, and the stress value changes were based on a varus angle of 0°, it was impossible to determine whether stress shielding or overloading could occur. The results of this study only indicate that stress shielding may occur when stress values decrease, and overloading may occur when stress values increase. Finally, it should be noted that the results of this study may not apply to all cases, as no analysis was performed on male patients.

Additional studies on stem flexion/extension, valgus, anterior/posterior insertion, and male patients will further clarify the advantages and disadvantages of short stems.

## Conclusion

Most studies only evaluated a group of cases of stem varus malalignment with periosteal reactions in imaging studies, and none investigated the stem varus angle. In the present study, stress decreased with increasing varus angle in zone 2 of the Gruen classification when the varus angle was ≥ 3°, suggesting the appearance of a grade 3 Engh classification of stress shielding and the appearance of cortical hypertrophy in zones 3 and 4. In the champagne-flute type (type A) with a narrow medullary cavity, a significant increase in distal stress was observed at a 2° varus angle, and careful stem insertion was necessary. Taperloc microplasty provided good distal load transfer in all femoral medullary configurations when the stem was neutrally inserted.

## Data Availability

No datasets were generated or analysed during the current study.

## Abbreviations

CFI: Canal Flare Index
CT: Computed tomography
THA: Total hip arthroplasty
VMS: Von Mises stress
