# Correction: Finite element analysis of mechanical stress in a cementless tapered-wedge short stem in the varus position

**DOI:** 10.1186/s13018-024-04945-z

**Published:** 2024-08-07

**Authors:** Takahiro Maeda, Osamu Obayashi, Muneaki Ishijima, Taichi Sato, Yoshiro Musha, Hiroyasu Ikegami

**Affiliations:** 1 Department of Orthopedic Surgery, Toho University Graduate School of Medicine, 5-21-16 Omorinishi, Ota-ku, Tokyo 143-8540 Japan; 2 Department of Orthopedic Surgery (Ohashi), School of Medicine, Toho University, 2-22-36 Ohashi, Meguro-ku, Tokyo 153-8515 Japan; 3 Department of Orthopedic Surgery, Juntendo Shizuoka Hospital, Nagaoka 1129, Izunokuni, 410-2295 Shizuoka Japan; 4 Department of Medicine for Orthopedic and Motor Organ, Juntendo University Graduate School of Medicine, 2-1-1, Hongo, Bunkyo-ku, Tokyo 113-8421 Japan; 5 Department of Advanced Machinery Engineering, School of Engineering, Tokyo Denki University, 5 Senju Asahi-cho, Adachi-ku, Tokyo 120-8551 Japan

**Correction: Journal of Orthopaedic Surgery and Research (2024) 19:385**

10.1186/s13018-024-04856-z

Following publication of the original article [1], an error was identified in the **Ethics approval and consent to participate** and **Consent for publication.**

The updated **Ethics approval and consent to participate** and **Consent for publication** are given below and the changes have been highlighted in **bold typeface**.

**Ethics approval and consent to participate**

The research protocol was approved by the Research Ethics Committee of the Faculty of Medicine, Juntendo University (research project number E21-0191), and research permission was obtained from Toho University Medical Center Ohashi Hospital (control NumberC_H21009).

**Information about the study was disclosed on the institutional website**,** and potential participants were given the opportunity to opt-out.**

Consent for publication

**Not applicable.**

The original article has been corrected.

