# Correction: Finite element analysis of mechanical stress in a cementless tapered-wedge short stem in the varus position

**DOI:** 10.1186/s13018-024-05307-5

**Published:** 2024-11-28

**Authors:** Takahiro Maeda, Osamu Obayashi, Muneaki Ishijima, Taichi Sato, Yoshiro Musha, Hiroyasu Ikegami

**Affiliations:** 1 Department of Orthopedic Surgery, Toho University Graduate School of Medicine, 5-21-16 Omorinishi, Ota-ku, Tokyo, 143-8540 Japan; 2 Department of Orthopedic Surgery (Ohashi), School of Medicine, Toho University, 2-22-36 Ohashi, Meguro-ku, Tokyo, 153-8515 Japan; 3 Department of Orthopedic Surgery, Juntendo Shizuoka Hospital, Nagaoka 1129, Izunokuni, Shizuoka 410-2295 Japan; 4 Department of Medicine for Orthopedic and Motor Organ, Juntendo University Graduate School of Medicine, 2-1-1, Hongo, Bunkyo-ku, Tokyo, 113-8421 Japan; 5 Department of Advanced Machinery Engineering, School of Engineering, Tokyo Denki University, 5 Senju Asahi-cho, Adachi-ku, Tokyo, 120-8551 Japan

**Correction: Journal of Orthopaedic Surgery and Research (2024) 19:385**

10.1186/s13018-024-04856-z

Following publication of the original article, an error was identified in the Fig. 2 caption.

The updated Fig. 2 caption is given below and the changes have been highlighted in **bold typeface**.

① The sentence currently reads:

Fig. 2 Analysis conditions. The load restraint conditions were set assum-ing a one-leg standing posture, with 2,400 N at the stem head from 15°proximal medial to the femoral axis and 1,200 N at the abductor pollicisbrevis from 20° distal lateral to the femoral axis

The sentence should read:

Fig. 2 Analysis conditions. The load restraint conditions were set assum-ing a one-leg standing posture, with 2,400 N at the stem head from 15°proximal medial to the femoral axis and 1,200 N at the **abductor muscles** from 20° distal lateral to the femoral axis

The original article has been corrected.

